# Clinical and biochemical determinants of length of stay, readmission and recurrence in patients admitted with diabetic ketoacidosis

**DOI:** 10.1080/07853890.2023.2175031

**Published:** 2023-02-06

**Authors:** Fateen Ata, Adeel Ahmad Khan, Ibrahim Khamees, Phool Iqbal, Zohaib Yousaf, Bayan Z. M. Mohammed, Reham Aboshdid, Sandy K. K. Marzouk, Haidar Barjas, Madiha Khalid, Ihab El Madhoun, Mohammed Bashir, Anand Kartha

**Affiliations:** a Department of Endocrinology, Hamad Medical Corporation, Doha, Qatar; b Department of Medicine, Hamad Medical Corporation, Doha, Qatar; c Department of Medicine, New York Medical College/Metropolitan Hospital Center, New York, NY, USA; d Department of Medicine, Reading Hospital-Tower Health, West Reading, PA, USA; e Department of Geriatrics, Hamad Medical Corporation, Doha, Qatar; f College of Medicine, Qatar University, Doha, Qatar; g Department of Nephrology, Hamad Medical Corporation, Doha, Qatar; h College of Medicine, Weill Cornell Medicine, Doha, Qatar

**Keywords:** Diabetic ketoacidosis, DKA, type 1 diabetes, type 2 diabetes, length of stay, recurrence, readmission

## Abstract

**Background:**

The increasing prevalence of diabetic ketoacidosis (DKA) related admissions poses a significant burden on the healthcare systems globally. However, data regarding the predictors of healthcare resource utilization in DKA is limited and inconsistent. This study aimed to identify key predictors of hospital length of stay (LOS), readmission and recurrent DKA episodes.

**Methods:**

We undertook a retrospective cross-sectional analysis of all DKA admissions from 2015 to 2021 across four hospitals in Qatar. The primary outcomes were the length of stay (LOS), 90-day readmission and 6-month and 1-year DKA recurrence.

**Results:**

We included 922 patients with a median age of 35 years (25–45). 62% were males with type-1 diabetes-mellitus (T1DM) and type-2 DM (T2DM), present in 52% and 48% of patients. The median LOS was 2.6 days (IQR 1.1–4.8), and the median DKA resolution time was 18 h (10.5–29). Male-gender, new-onset DM, higher Charlson Comorbidity Index (CCI), lower haemoglobin, sodium and potassium, higher urea, longer DKA duration and MICU admission predicted a longer LOS in a multivariate regression analysis. None of the factors were significantly associated with 90-day readmission. Patients with pre-existing T1DM were more likely to have a six-month DKA recurrence than pre-existing T2DM. Patients with a 6-month DKA recurrence, female gender and T1DM had higher odds of 12-month recurrence, whereas a consult with a diabetes educator at the index admission was associated with decreased odds of recurrence.

**Conclusions/interpretation:**

This is the most extensive study from the Middle-East region reporting on LOS, readmissions and the recurrence of DKA. Results from this study with a diverse population may be valuable for physicians and healthcare systems to decrease the diabetes-related healthcare burden in DKA patients.

## Graphical Abstract

**Figure:**
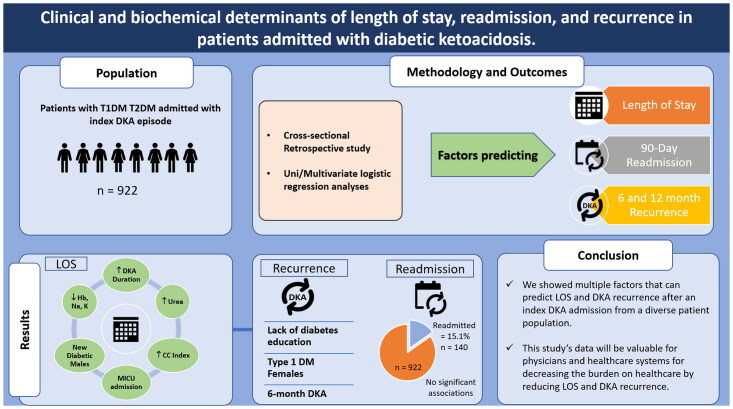

**Key Messages**
- This study showed that the hospital length of stay and recurrent episodes can be predicted in patients with diabetic ketoacidosis (DKA).
- Multimorbidity, male gender, newly developed T2DM, anaemia, hyponatremia, hypokalaemia, high urea levels and intensive care can predict LOS in DKA patients.
- DKA recurrence at 6 and 12 months is higher in females, younger age, non-obese patients, poor insulin compliance and in pre-existing T1DM.

## Introduction

Diabetic ketoacidosis (DKA) is an acute, potentially fatal complication of DM. Hospitalizations secondary to DKA are seeing an increasing trend [1]. It is a leading cause of mortality in children and young adults with type 1 diabetes (T1DM), causing around 50% of deaths [2]. Furthermore, DKA is a significant healthcare burden, with more than 500,000 annual hospital admissions in the USA and an annual cost of more than 2.4 billion USD [3]. The incidence rate of DKA is variable, ranging between 20 per 1000 person-years (PY) in North America to 27–51 per 1000 PY in Europe [4]. Qatar has the fourth-highest incidence of T1DM [5]. It is witnessing a rising trend of T1DM and T2DM cases, with prevalence reaching up to 17% [6]. With rising DM, DKA is expected to become one of the most significant healthcare concerns in the region. However, the literature lacks regional data on the factors that might increase healthcare resource utilization in DKA.

Although mortality related to DKA is decreasing, admissions and healthcare burden continues to rise. Desai et al. showed that inflation-adjusted healthcare costs for DKA-related admissions increased from 2.2 billion USD in 2003 to 5.2 billion USD in 2014 [3]. However, factors affecting healthcare-related costs and patient outcomes in DKA are unclear. Reducing the length of stay is associated with cost-cutting and better patient outcomes. Ahmann et al. showed a reduction of $2,353 per patient secondary to a reduced hospital stay in patients with diabetes [7]. Whether a reduction of already short hospital stay in DKA significantly reduces healthcare cost is an underexplored aspect. One study from the USA showed increased medical bills despite a reduced LOS among DKA patients [3]. Whereas another nationwide analysis showed reduced hospitalization cost (which is partly reflected by lower LOS) as a marker of resource utilization in DKA hospitalizations [8]. DKA protocols have shown a well-evident decrease in LOS among admitted patients with DKA [9,10]. Most healthcare systems now use DKA protocols for effective management. However, other factors that can impact LOS, readmissions, and recurrences have yet to be evaluated in similarly sufficient detail.

Multiple factors have been linked to the increased LOS in DKA. Freire et al. studied 584 DKA patients and showed that only the triggering factors of DKA are significantly associated with LOS [11]. Another study linked DKA severity with prolonged LOS [12]. Another study illustrated that new-onset DM is the most critical factor for prolonged LOS among 500 DKA episodes [13]. Other factors linked to prognosis and recurrence in DKA are patient demographics such as age, albumin levels, lactate clearance, hyperchloremia, platelet-lymphocyte ratios, and compliance with insulin [14–21]. This study aims to identify factors that can predict LOS, readmission, and recurrence in a large and diverse patient population, which may provide more robust and generalizable results concerning healthcare resource utilization in DKA.

## Research design and methods

### Study design

We performed a retrospective data review on all patients admitted with DKA from January 2015 to March 2021 in Hamad Medical Corporation, which provides most of the tertiary acute healthcare in the nation of Qatar.

### Ethical approval

Ethical approval for this study was obtained from the institutional review board at the medical research centre (MRC), Qatar (MRC-01-21-476). Informed consent was waived, given the study’s retrospective design with anonymized data collection and analysis.

### Inclusion criteria and data collection

We identified patients with index DKA admissions between 2015 and 2021 to four hospitals of Hamad Medical Corporation, Qatar (Hamad General Hospital, Al-Khor Hospital, Al-Wakra Hospital, and Hazam Mebaireek General Hospital) through electronic health records using the International Classification of Diseases (ICD) coding for DKA.

The inclusion criteria were adult patients (>14 years of age) with a known diagnosis (or a new diagnosis on the index DKA admission) of T1DM or T2DM based on glycated haemoglobin (HbA1c) ≥6.5% or a fasting glucose ≥7.0 mmol/L at the time of DKA diagnosis, either at admission or before the relevant admission. The ketoacidosis diagnosis was confirmed if the patients had: pH <7.3, anion gap >10 mmol/L, and ketonemia/ketonuria. Glucose level was not used to define DKA cases to include euglycemic DKA patients in the study cohort. We excluded patients with other causes of ketoacidosis, such as starvation and alcohol-induced ketoacidosis. American diabetes association classification of DKA severity was used to categorize patients into mild, moderate, and severe DKA [22]. DKA was defined as mild if the patient had an initial PH of 7.25–7.30 or initial serum bicarbonate level of 15–18 mmol/L, moderate if the initial PH of 7.00 to <7.24 or the initial serum bicarbonate level of 10 to <15 mmol/L, and severe if the PH <7 or initial serum bicarbonate level of <10 mmol/L.

Data of the included patients were extracted from the electronic records of the Hamad Medical Corporation patient data repository (Cerner). Data collected includes demographics such as age, sex, ethnicity, body mass index (BMI), comorbid conditions, and relevant laboratory investigations at admission. Charlson Comorbidity Index (CCI) was utilized to quantify the comorbidity burden in the patient population [23].

Data relevant to patients’ diabetes status included HBA1C recorded on or within the last three months of presentation, medication compliance, and diabetes complications. Data collected for DKA includes relevant laboratory investigations such as complete blood counts, electrolyte panels, serial PH, anion gap, lactic acid, glucose, blood/urine ketones, possible triggering factors, DKA duration, hospital days, need for MICU admission, in-hospital mortality, 90-day readmission with any cause, 6-month and 12-month recurrence.

### Study outcomes

The primary outcome of our study was the length of stay in patients with index admissions of DKA. Secondary outcomes included 90-day all-cause readmission, 6-month recurrence with DKA, and 12-month recurrence with DKA. We chose 90-day all-cause as it is one of the strongest risk factors for readmission in patients with diabetes [24]. A recurrence of DKA at 6 and 12 months was selected because the literature shows significantly higher odds of recurrence at 6 months and the second year of the index DKA admission [25,26].

### Statistical analyses

We used descriptive statistics to present the demographic data of the study cohort. We classified DM as either type 1 or type 2 DM, and whether it was now onset (diagnosed at the index DKA admission) or pre-existing. We categorized the cohort into four ethnic groups: Arab, Asian, African and others. Ethnic-specific cut-off points were used to categorize BMI into normal, overweight, and obese. We classified patients into high metabolic risk based on the presence of one or more of the following factors: obesity, hypertension, and dyslipidemia. Continuous variables were summarized as mean (SD) and median (IQR), while categorical variables were summarized as percentages. We compared continuous parametric variables using unpaired t-test and ANOVA. Man-Whitney U test and Kruskal–Wallis tests were used to compare non-parametric continuous data. We used the Chi-square test to compare categorical variables. Univariate analysis was performed for the dependent variables, including LOS, 90-day readmission and 6 and 12 months DKA recurrence. The multiple logistic models included all independent variables with a *p* value ≤.1 at the univariate level. The length of hospital stay was analysed using a multivariate linear regression model, whereas we used a multivariate logistic regression model for analysing DKA recurrence. We used STATA 15 for the analysis.

## Results

**Figure 1.**
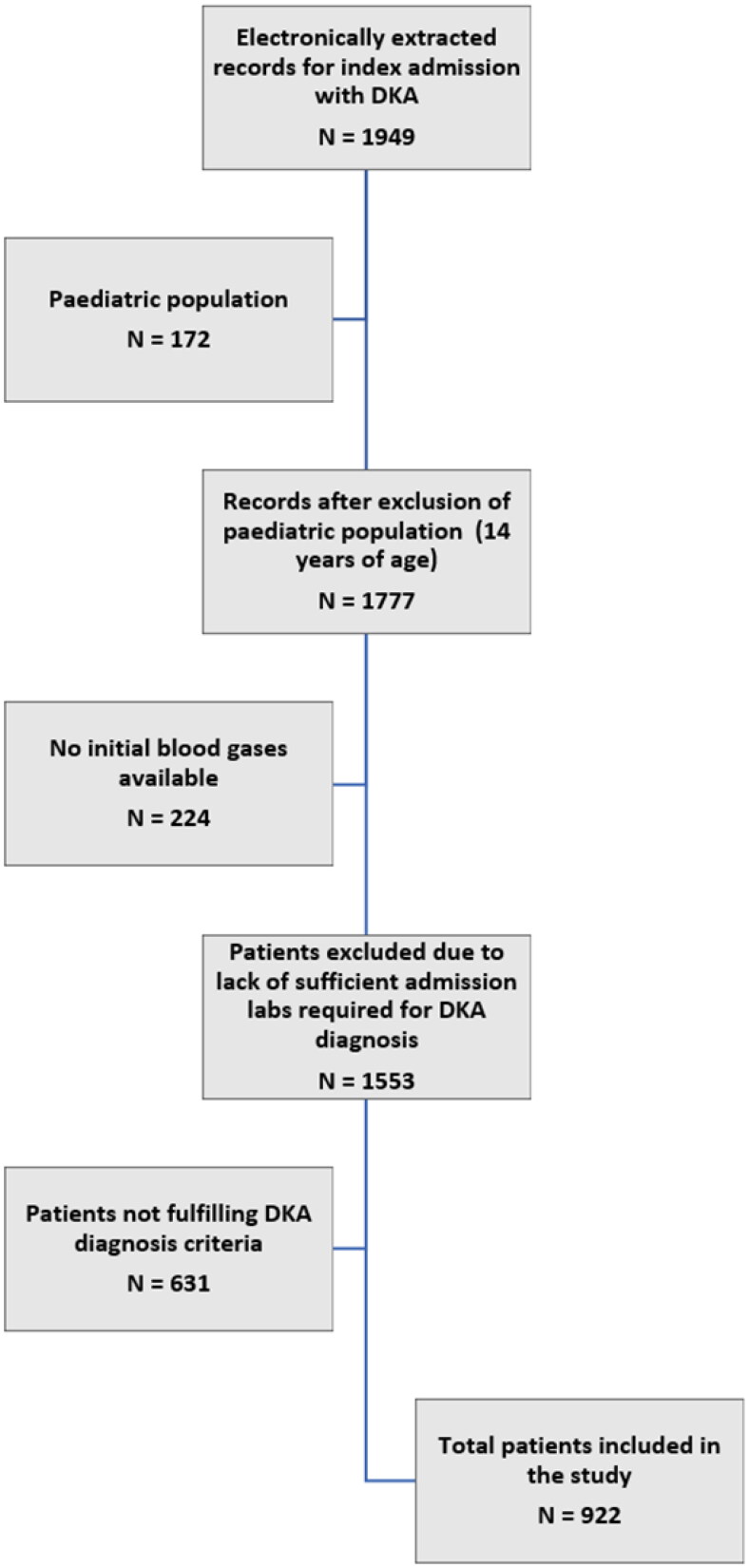
Flow chart of the process of inclusion of patients in the study.

A total of 1949 consecutive patients with index DKA admissions (identified through electronic medical records) were assessed for eligibility (from 1 January 2015 to 1 March 2021). After applying the inclusion/exclusion criteria, 922 patients were added to the study and the final analysis (Figure 1).

### Clinical characteristics of the study population

The baseline characteristics of the study population are summarized in Table 1 and Supplementary Table 1. The median age of the patients was 35 years (IQR 25–45), out of which 575 (62%) were males. The mean body mass index (BMI) was 24.8 ± 6.2 kg/m^2^. Most patients had a normal BMI (18.5–25) kg/m^2^. (*N* = 475, 51.9%). The median duration of DM was six years (IQR 3–12). Among these, 480 (52%) had T1DM, whereas 442 (48%) had T2DM.

**Table 1.** Characteristics of the patients admitted with index DKA episode.

Baseline characteristics	Results (*N* = 922)	
Age in years, median (IQR)	35 (25–45)	
Males (%)	575 (62.4%)	
Ethnicities, N (%)	Arabs	502 (54.4%)
	Asians	300 (32.5%)
	Africans	90 (9.7%)
	Others	30 (3.2%)
T1DM	Pre-existing	332 (36%)
	New	148 (16%)
	Total	480 (52%)
T2 DM	Pre-existing	266 (28.8%)
	New	176 (19%)
	Total	442 (47.9)
Comorbidities	Microvascular complications	95 (10.3%)
	Macrovascular complications	56 (6%)
	High metabolic risk	394 (42.7%)
HBA1c on admission, mean (SD	12.1 ± 2.7 %	

*Note:* BMI: body mass index; DM: diabetes mellitus; T1DM: type 1 diabetes; T2DM: type 2 diabetes; COPD: chronic obstructive pulmonary disease; metabolic risk: dyslipidemia, ethnic-specific obesity, hypertension.

**Figure 2.**
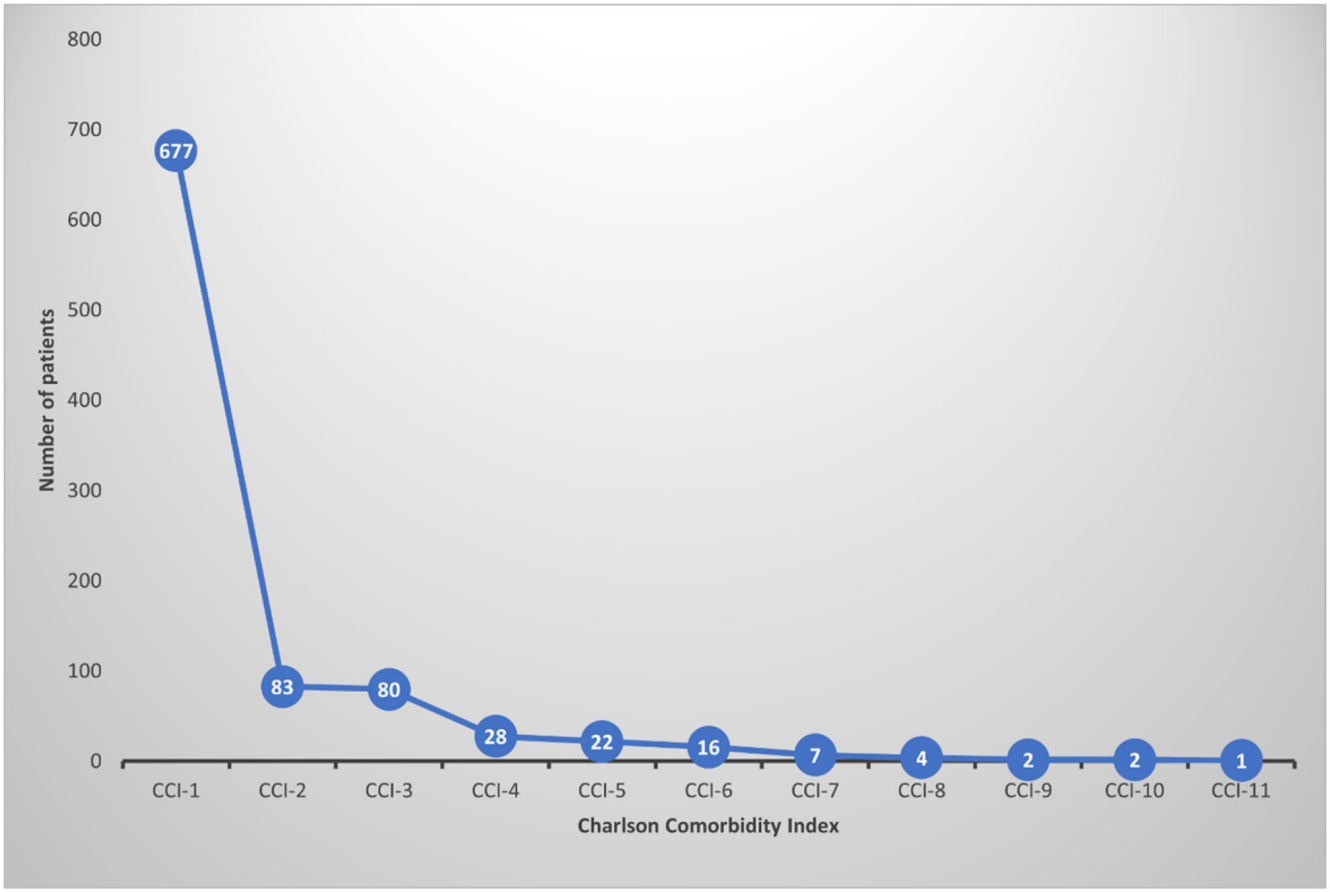
Charlson Comorbidity Index showing the comorbidity status of the study cohort.

We used CCI to assess the comorbidity burden [23]. The majority had a low CCI (73.4% belonging to CCI 1) (Figure 2). Microvascular and macrovascular diabetes complications were identified in a minority of patients (10.3% and 6%, respectively). Metabolic risks (dyslipidemia, ethnicity-specific obesity, hypertension) were identified in 394 (42.7%) patients. Interestingly, the largest proportion of patients had severe DKA (42%).

**Figure 3.**
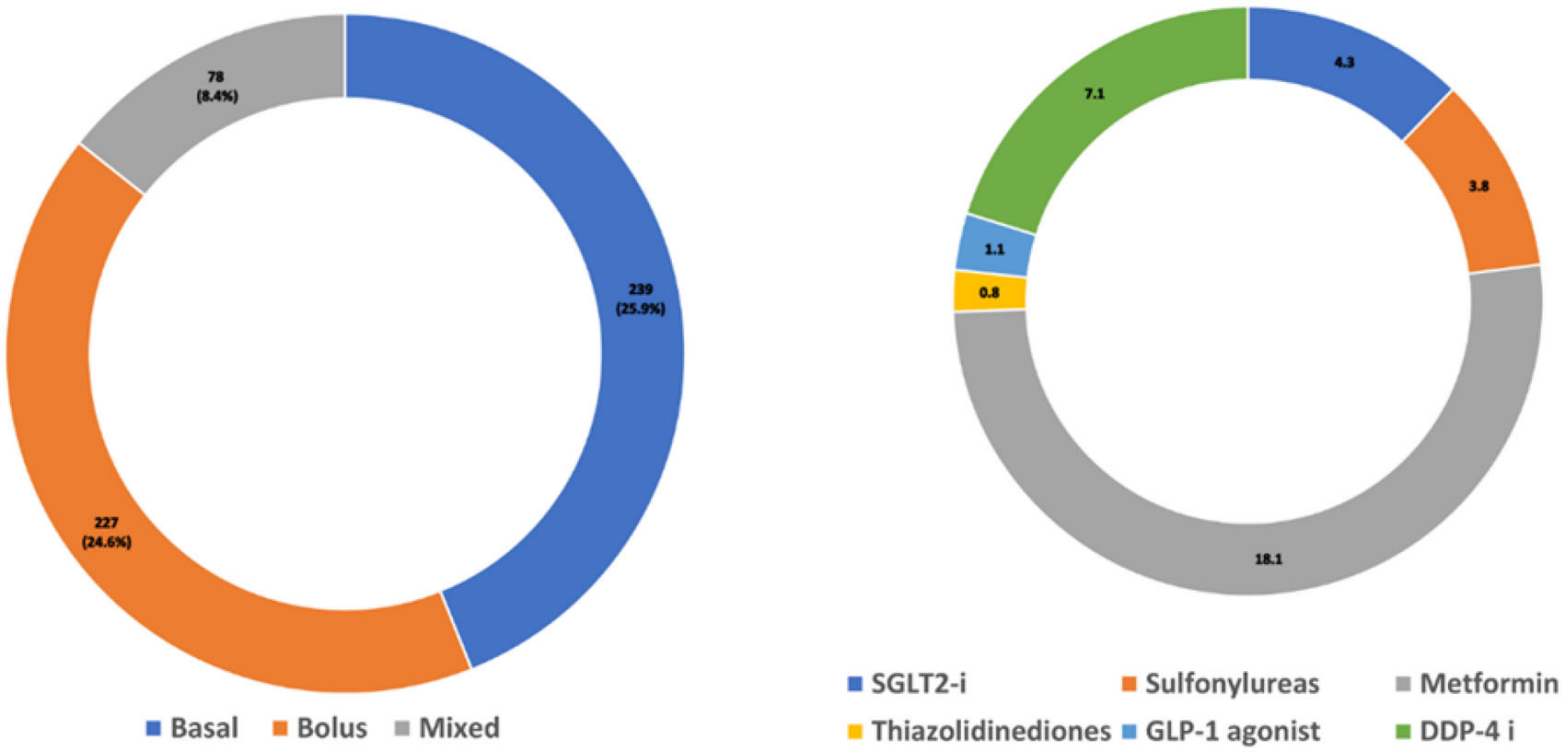
Frequency of diabetes related medication use among the study cohort. (3a) Use of insulin types. (3b) Use of oral anti-diabetic medicines.

**Figure 4.**
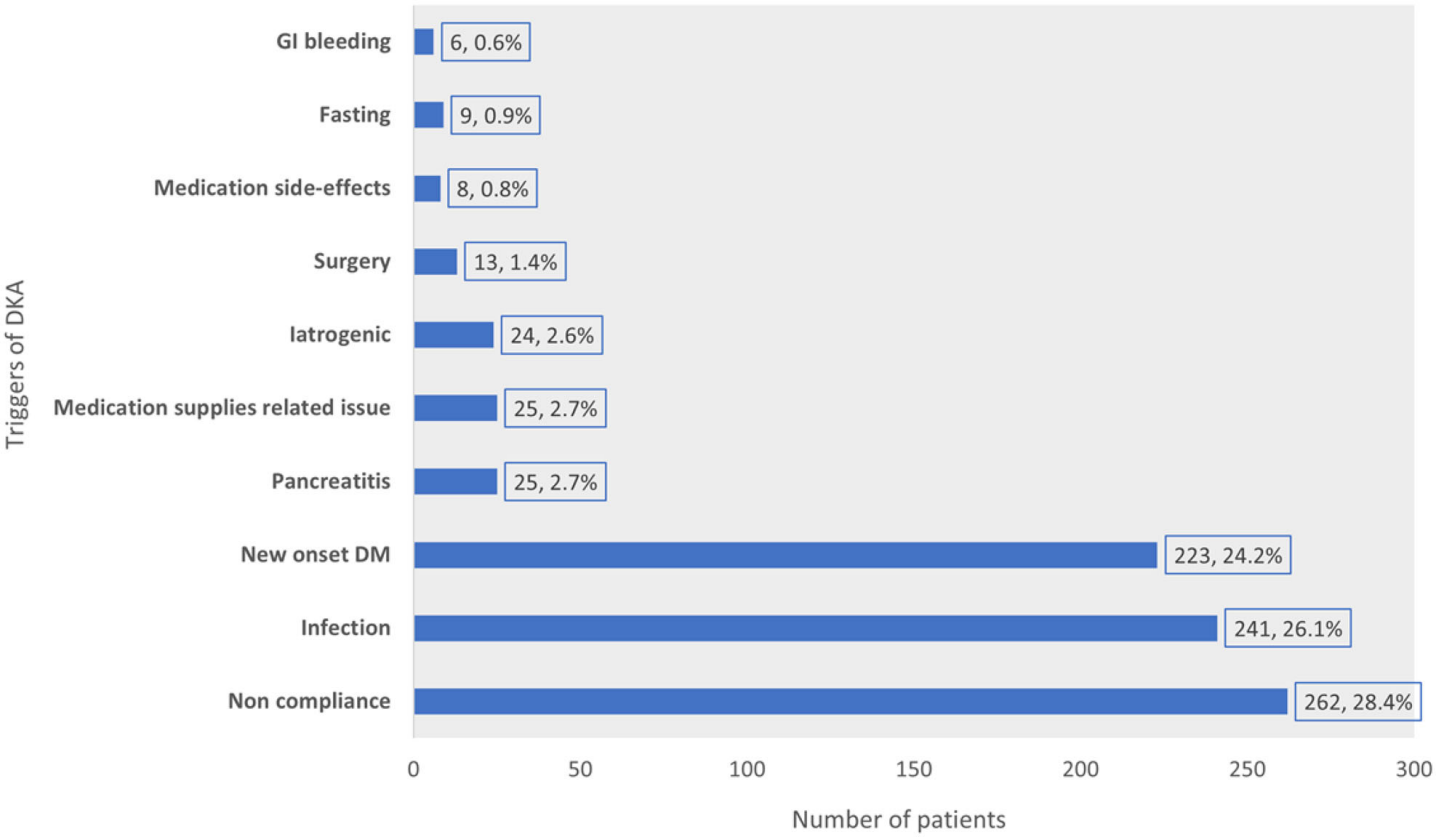
Reported triggering factors of DKA development during the index admission in the study cohort (iatrogenic: inadequate medication dosage prescribed, medications side-effects: DKA secondary to SGLT2 inhibitors, DDP-4 inhibitors and GLP-1 agonists).

The median insulin dosage was 0.9 (IQR 0.7–1.1) units/kg/day. The mean dosage in T1DM was 1.07 ± 0.4 units/kg/day, whereas, in T2DM, it was 0.8 ± 0.4 units/kg/day. Compliance with insulin was reported at 32.2%. Variable use of basal, bolus and mixed insulin was reported in the study cohort (Figure 3(a)). Among the oral medications, metformin was the most commonly used anti-diabetic agent (18.1%) (Figure 3(b)). We identified various triggering factors of DKA from the admission charts. The most common trigger of DKA was non-compliance. Triggering factors are summarized in Figure 4. Of the 480 patients with T1D, diabetes educators (DE) were consulted in 128 (26.7%), whereas in the 442 patients with T2D, DE consultation was reported in 49.7% (*p* < .001). Older patients were consulted more with DE compared to younger patients (OR 1.02, CI: 1.01–1.03, *p* < .001).

### Laboratory findings

Among the general haematology investigations, the median white blood cell count (WBC) was high (12 (8.3–17.4) × 10^^3^/uL), with a predominance of absolute neutrophil count (ANC) (10.6 ± 6.4 × 10^^3^/uL). The mean HbA1c at admission was elevated (12.05% ± 2.7) (Table 1). Other admission laboratory investigations and sequential blood gas results recorded at admission, 2-h, 4-h and 6-h are tabulated (Supplementary Tables 2 and 3).

### Clinical outcomes

#### Length of stay

The median length of stay was 2.6 (IQR 1.1–4.8) days, whereas the median time to DKA resolution was 18 (IQR 10.5–29) h. A considerable proportion of the patients (24.8%) required medical intensive care unit (MICU) admission. Overall, in-hospital mortality was low (0.7%) (Table 2).

**Table 2.** Clinical outcomes of patients admitted with index DKA episodes (*N* = 922).

Outcomes	Results
LOS in days, median (IQR)	2.6 (1.1–4.8)
DKA duration in hours, median (IQR)	18 (10.5–29)
DKA severity, N (%)	
Mild	202 (21.9%)
Moderate	332 (36%)
Severe	388 (42%)
Inpatient GI bleed, N (%)	7 (0.7%)
COVID-19 infection, N (%)	11 (1.19%)
Inpatient NSTEMI, N (%)	9 (0.9%)
In-hospital mortality, N (%)	7 (0.7%)
Need for admission to MICU, N (%)	229 (24.8%)
Consult to a diabetes educator, N (%)	348 (37.7%)
DKA recurrence, N (%)	
6 months	73 (7.9%)
12 Months	61 (6.6%)
90-Day all-cause readmission	140 (15.1%)

*Note:* DKA: diabetic ketoacidosis; LOS: length of stay; GI: gastrointestinal; COVID-19: coronavirus disease 2019; MICU: medical intensive care unit.

Univariate analysis showed male gender (*p* value <.001), Asian ethnicity (*p* value <.001), T2DM (*p* value <.001), presence of metabolic risks (dyslipidemia, ethnic-specific obesity, hypertension) (*p* value <.001), microvascular complications (*p* value .01), macrovascular complications (*p* value <.001) were associated with a significantly longer LOS. Among the triggering factors of DKA, gastrointestinal bleeding and pancreatitis were associated with a significantly longer LOS. Lastly, severe DKA was associated with a significantly longer LOS compared to mild and moderate DKA (*p* value .002). A complete univariate analysis is reported in Supplementary Table 4.

A multivariate linear regression analysis model was generated with clinically and statistically significant variables to identify the predictors of a longer length of stay in our cohort. Higher CCI (*p* value .03), lower haemoglobin (Hgb) at admission (*p* value .005), higher urea at admission (*p* value <.001), lowest sodium during hospital stay (*p* value .01), lowest potassium (*p* value .02), male gender (*p* value .002), new diabetes (*p* value .03), longer DKA duration (*p* value <.001) and MICU admission (*p* value <.001) remained significant factors predicting a prolonged LOS in patients admitted with DKA (Table 3).

**Table 3.** Multivariate linear regression analysis model for the factors predicting length of stay.

Variable	Co-efficient	t	Significance	95% confidence interval	
				Lower bound	Upper bound
CCI	0.56	2.14	0.03	0.04	1.08
Hgb at admission	−0.39	−2.84	0.005	−0.67	−0.12
Urea at admission	0.25	4.48	<0.001	0.14	0.36
Lowest sodium	−0.13	−2.58	0.01	−0.24	−0.33
Lowest potassium	−1.42	−2.23	0.02	−2.67	−0.17
Female gender	−1.93	−3.14	0.002	−3.14	−0.7
New DM	1.19	2.09	0.03	0.07	2.31
DKA duration (hours)	0.05	3.71	<0.001	0.02	0.08
MICU admission	2.59	4.6	<0.001	1.48	3.7

*Notes:* Corrected for age, N/L ratio, type of DM, creatinine, metabolic risks and DKA severity (*N* = 922). MICU: medical intensive care unit; DM: diabetes mellitus; N/L: neutrophil/lymphocyte ratio; Hgb: haemoglobin; CCI: Charlson Comorbidity Index; DKA: diabetic ketoacidosis.

#### 90-Day readmission

One hundred forty patients (15%) were readmitted within 90 days (due to any cause). Univariate regression analysis of the baseline characteristics and laboratory investigations at discharge could not identify any significant predictors (Supplementary Table 5).

#### Recurrence of DKA at 6 and 12 months after the index episode

Recurrence of DKA admissions at 6 months and 12 months from the index DKA episode was recorded in 7.9% and 6.6% of patients, respectively. Pre-existing T1DM were more likely to have six-month DKA recurrence compared to pre-existing T2DM (*p* value <.001). No other factor showed any significant associations with recurrence at six months (Supplementary Table 6). Multiple factors were associated with a 12-month DKA recurrence from the index admission. These included younger age (*p* value .001), female gender (*p* value <.001), Arabic ethnicity (*p* value .001), pre-existing T1DM (*p* value <.001), lower weight (*p* value .04), insulin non-compliance (*p* value .001), shorter LOS at index admission (*p* value .02) and a recurrence of DKA at six months (*p* value <.001) (Supplementary Table 7). In a multivariate logistic regression, 6-month DKA recurrence (OR 54.35, *p* value <.001) and female gender (OR: 2.58, *p* value .01) increased the risk of 12-month recurrence, whereas T2DM had a lower risk of recurrence at 12 months compared to T1DM (OR 0.3, *p* value .02). Lastly, patients whom Diabetes educators (DE) had seen during the index admission had a lower risk of 12-month recurrence (OR 0.3, *p* value .02) (Table 4).

**Table 4.** Multivariate logistic regression analysis model with predictors of 12-month recurrence. Corrected for age, length of stay and ethnicity (*N* = 922).

Variable	Odds ratio	95% confidence interval		Significance
		Lower bound	Upper bound	
6-month recurrence	54.35	26.12	113.1	<0.001
Consult to DE	0.3	0.13	0.86	0.02
T2DM	0.3	0.10	0.85	0.02
Female gender	2.58	1.25	5.34	0.01

*Note:* T2DM: type 2 diabetes; DE: diabetes educators.

## Discussion

In this study, we have investigated prognostic factors of DKA with an in-depth analysis of possible predictors of LOS, readmission, and recurrence after an index DKA admission in 922 patients with diabetes from the main tertiary referring hospitals in the state of Qatar. The median LOS was 2.6 days (IQR 1.1–4.8). 90-Day all-cause readmission occurred in 15.1%. A 6-month DKA recurrence was seen in 7.9% of the three patients, whereas (6.6%) had a recurrence at 12 months. Most patients had a severe DKA at the index admission (42%), whereas moderate and mild DKA was observed in 36% and 21.9% of the patients, respectively. MICU admission was reported in 24.8%, and 0.7% of the patients died within the index admission. We identified multimorbidity, male gender, newly developed T2DM, anaemia, hyponatremia, hypokalaemia, high urea levels, and MICU admission as strong predicting factors of increased LOS in DKA patients. On the other hand, the DKA recurrence at 6 and 12 months was higher in females, younger age, non-obese, poor insulin compliance, and in pre-existing T1DM. Additionally, a review by a diabetes educator during the index admission was associated with decreased odds of DKA recurrence at 12 months.

CCI is a well-acknowledged assessment tool for patients’ health status and hospital-related outcomes [27]. We demonstrated that a higher comorbidity burden is significantly associated with longer LOS in DKA patients. Numerous studies linked a high comorbidity burden with the development of DKA; however, data are limited regarding the effect of a high comorbidity burden on LOS in DKA admissions [28]. A recent study published in 2022 also reported an association of higher CCI with prolonged LOS in DKA patients [8]. It has shown clinical significance in predicting various diseases’ short-term and long-term clinical outcomes [29]. Its utility in predicting LOS and recurrence in patients with DKA might significantly aid in better management of the patients.

Farooq et al. recently (February 2022) studied the effect of acid suppression with a prolonged LOS in a large cohort of DKA patients. Although they mainly studied the effect of peptic ulcer disease on DKA, they also analysed some factors affecting the LOS common to our data [8]. These included age, gender, and metabolic risks. Although metabolic risks, male gender, and lower age were associated with a longer LOS in this study, the association could not be retained in the multivariate regression. This variation in results can be due to the difference in the study population; a predominantly male multi-ethnic younger population in this study compared to a relatively older population with a balanced gender distribution [8]. Other than the events of index admission, readmission with any cause and DKA recurrence is clinically meaningful and commonly encountered outcomes associated with an increased healthcare burden and poor patient outcomes. Young females are thought to be at high risk of short-term (30-day) DKA recurrence, as evidenced by literature [14,30]. The current study further consolidates that patients who are young are at higher risk of long-term (6 and 12-month) DKA recurrence in addition to female gender. In addition, similar to this study, previous studies have also shown that patients with more than one DKA episode have stronger odds of further recurrent DKA episodes [31]. Diabetes educators (DE) have an instrumental role in improving patient outcomes related to DM [32]. Consult with DE during the index admission in this study was associated with a lower risk of DKA. Hence, ensuring adequate diabetes education in all patients admitted with DKA is a critical intervention to reduce recurrence.

Data from several studies have identified young patients with diabetes as more prone to medication non-adherence, especially insulin [33–35]. This translates into an increased risk of developing DKA, or its recurrence, as shown in this study. Similarly, it is evident from the literature that young patients with diabetes tend to have a longer LOS in DKA admissions [8]. Therefore, young patients with diabetes, mainly newly diagnosed, should be the target population to address the rising healthcare resource utilization issues concerning DKA. Factors such as peer pressure causing non-adherence to treatment, lack of a clear insight into the consequences of non-compliance, and forgetfulness to take medications need to be addressed systematically to decrease the recurrence of DKA and minimize the healthcare resource utilization once admitted with DKA. Public awareness of precipitators of DKA and preventive techniques, as well as individualized education through DE if admitted with DKA, are central to resource rationalization and better patient outcomes. Education on managing diabetes during sick days is an effective measure of reducing the risk of DKA. The latest consensus guidelines on sick day rules in managing diabetes in young adults focus on utilizing every opportunity in re-education [36]. Thorough education on sick day rules before discharge in index DKA admissions can significantly improve patient and healthcare outcomes.

The principal strength of this study lies in its large and diverse cohort and an in-depth analysis of multiple factors that can predict healthcare resource utilization in terms of LOS, readmission, and recurrences in patients with DKA. The many ethnicities represented in this study population strengthen the generalizability of our findings to these populations and the Middle East region. We applied the American diabetes association DKA diagnostic criteria on the initial electronically extracted records to exclude those patients who had wrong coding based on the diagnostic criteria, allowing us to present a more valid patient population admitted with DKA. Our study has some limitations, most importantly it being a retrospective rather than a prospective data analysis. Hence, cofounders might have some influence on the results. Additionally, there might have been some patients whose diagnosis was not coded as DKA in the EMR and were not included in the study. Although theoretically, these patients may have had different outcomes than the study cohort, their number is expected to be much lower than the study cohort size; hence the influence on results would be minimal at best.

## Conclusion

This study highlights various clinical and biochemical factors associated with prolonged LOS and recurrence of DKA in a sizeable cohort of patients with diabetes. LOS, readmissions, and recurrence of preventable disease processes are outcomes of interest in the current area where healthcare has seen global exhaustion amid the ongoing pandemic. Efforts to reduce this burden in DKA patients will not only improve the prognosis of their diabetes but will also aid the healthcare setups to focus better on other non-preventable admissions. Data from this study will be valuable for treating physicians and administrators to decrease the healthcare burden by reducing LOS and recurrence in DKA patients. Additionally, future research can focus on these significant factors in building scoring systems for a more practical prediction of LOS and recurrence in DKA patients.

## Supplementary Material

Supplemental Material

Supplemental Material

## Data Availability

Available from the corresponding author upon reasonable request.
