# Supplementary material for: Clinical and biochemical determinants of length of stay, readmission and recurrence in patients admitted with diabetic ketoacidosis
Source: Ann Med. 2023 Feb 6;55(1):533–42. doi: 10.1080/07853890.2023.2175031 (PMC9904305; doi:10.1080/07853890.2023.2175031)
Supplement: Supplemental Material [file IANN_A_2175031_SM3429.docx]

**Supplementary Table 1.** Detailed demographics of the study cohort.

| **Baseline characteristics** | **Results** |
| --- | --- |
| **Ethnicity-adjusted BMI,** Mean (±SD) kg/m^2^ | 24.8 +/- 6.2 |
| Duration of DM in years, Median (IQR) | 6 (3-12) |
| **Co-morbidities**, N (%)  Dyslipidaemia  Depression  Schizophrenia  Bipolar disorder  Generalized anxiety disorder  Coronary artery disease  Heart failure  Peripheral vascular disease  Dementia  COPD  Asthma  Chronic liver Disease  Hypertension  Retinopathy  Nephropathy  Diabetic Foot  Amputation  Hemiplegia  Solid tumour  Lymphoma  HIV  Pregnancy | 128 (13.8)  30 (3.2%)  6 (0.6%)  3 (0.3%)  6 (0.6%)  56 (6%)  12 (1.3%)  6 (0.6%)  8 (0.8%)  64 (6.9%)  55 (5.9%)  34 (3.6%)  199 (21.5%)  78 (8.4%)  60 (6.5%)  25 (2.7%)  2 (0.2%)  6 (0.6%)  7 (0.7%)  1 (0.1%)  1 (0.1%)  10 (1%) |

(BMI: Body mass index, COPD: Chronic obstructive pulmonary disease, HIV: Human immunodeficiency virus)

**Supplementary Table 2.** Laboratory investigations of the study cohort at admission

| Laboratory investigations | Results |
| --- | --- |
| HbA1c, (<6.5 %), (Mean +/- SD) | 12.05 +/- 2.7 |
| RBG, mmol/L, Median (IQR) | 23.3 (18.2-30) |
| WBC, (4-10 10^3/uL), Median (IQR) | 12 (8.3-17.4) |
| Hgb, (13-17g/dl), Median (IQR) | 14.3 (12.9-15.7) |
| Plt , (150-410 10^3/uL), (Mean +/- SD) | 308.7 +/- 107.5 |
| ANC, (2-710^3/uL), (Mean +/- SD) | 10.6 +/- 6.4 |
| LC, (2-7 10^3/uL), (Mean +/- SD) | 1.8 +/- 0.9 |
| Plt/LC ratio, Median (IQR) | 180 (118.6 – 276) |
| ANC/LC ratio, Median (IQR) | 6.25 (2.7-11.4) |
| Urea, mmol/L, Median (IQR) | 5.8 (4.1-8.5) |
| Creatinine, umol/L, Median (IQR) | 87 (65-124) |
| CrCl , ml/min, Median (IQR) | 84.9 (61.4-124.6) |
| Sodium, mmol/L, Median (IQR) | 133 (130-136) |
| Potassium, mmol/L, Median (IQR) | 4.4 (3.9-5.0) |
| Chloride, mmol/L, Median (IQR) | 99 (95-104) |
| Phosphorus, mmol/L, Median (IQR) | 0.7 (0.3-1.0) |
| Albumin, g/L, Median (IQR) | 39.3 (33.9-44) |
| Osmolality, mmol/kg, Median (IQR) | 309 (300-321) |
| BHB, mmol/L, Median (IQR) | 5.8 (4.5-7.3) |
| CRP, mg/L, Median (IQR) | 18.6 (5-39) |
| Urine MAlb/Cr ratio, Mg/mmol, Median (IQR) | 2.3 (1.4-5.0) |
| Lactate, mmol/L, Median (IQR) | 1.7 (1.1-2.8) |
| Serum pH, Median (IQR) | 7.19 (7.08-7.27) |
| Bicarbonate, mmol/L, Median (IQR) | 11 (7.1-14.7) |
| Anion Gap, Median (IQR) | 22 (18-27) |

(HbA1c: Glycated haemoglobin, RBG: Random blood glucose, WBC: White blood cell count, Hgb: Haemoglobin, Plt: Platelets, ANC: Absolute neutrophil count, LC: lymphocyte count, CrCl: Creatinine clearance, BHB: Beta-hydroxybutyrate, CRP: C-reactive protein, MAlb/Cr: Microalbumin/creatinine)

**Supplementary Table 3.** Biochemical evolution of DKA of the study cohort during the index admission.

| Baseline characteristics | Results |
| --- | --- |
| Blood glucose at 2-hour, mmol/L, Median (IQR) | 16.4 (11.7-22.1) |
| Blood glucose at 4-hour, mmol/L, Median (IQR) | 14 (11-17.9) |
| Blood glucose at 6-hour, mmol/L, Median (IQR) | 12.2 (9.3-15.4) |
| Lactate at 2-hour, mmol/L, Median (IQR) | 1.9 (1.1-6.9) |
| Lactate at 4-hour, mmol/L, Median (IQR) | 1.55 (0.9-4.0) |
| Lactate at 6-hour, mmol/L, Median (IQR) | 1.3 (0.9-5.4) |
| Serum pH at 2-hour, Median (IQR) | 7.16 (7.04-7.2) |
| Serum pH at 4-hour, Median (IQR) | 7.2 (7.2-7.3) |
| Serum pH at 6-hour, Median (IQR) | 7.26 (7.1-7.3) |
| Bicarbonate at 2-hour, mmol/L, Median (IQR) | 9 (7-13.8) |
| Bicarbonate at 4-hour, mmol/L, Median (IQR) | 11.8 (7.4-15.7) |
| Bicarbonate at 6-hour, mmol/L, Median (IQR) | 15.4 (11.5-18.9) |

**Supplementary Table 4.** Univariate analysis of the factors associated with length of stay in patients with index DKA admissions

| Characteristics (N) | LOS (days) | p-value |
| --- | --- | --- |
| Age | - | <0.001 |
| Male  Female | 2.9 (1.4-5.3)  1.9 (1.0-4.0) | <0.001 |
| Ethnicity  Arabic  Asian  African  others | 1.9 (1-3.9)  3.6 (1.9-6.1)  2.7 (1.4-4.7)  2.3 (1.1-4.2) | <0.001 |
| DM diagnoses  Total T1D  Pre-existing T1D  New T1D  Total T2D  Pre-existing T2D  New T2D | 1.7 (1-3.4)  1.4 (1-2.7)  2.9 (1.4-4.9)  3.6 (1.8-6.3)  3.25 (1.7-5.7)  4 (2.1-7.6) | <0.001 |
| Dyslipidaemia  Absent  Present | 2.35 (1.1-4.7)  3.6 (1.8-6) | <0.001 |
| BMI  Category 1  Category 2  Category 3 | 2 (1-3.8)  2.6 (1.3-5.1)  3.9 (1.6-6.5) | <0.001 |
| Hypertension  Absent  Present | 2.2 (1-4.3)  3.6 (1.8-6.8) | <0.001 |
| Metabolic risks Absent  Present | 1.9 (1-3.9)  3.5 (1.7-6.1) | <0.001 |
| Retinopathy  Absent  Present | 2.5 (1.1-4.8)  2.9 (1.2-4.9) | 0.4 |
| Nephropathy  Absent  Present | 2.5 (1.1-4.6)  4 (1.9-9) | <0.001 |
| Microvascular complications  Absent  Present | 2.5 (1.1-4.7)  3.3 91.6-7) | 0.01 |
| CVA  Absent  Present | 2.5 (1.1-4.7)  5.4 (3.9.3) | <0.001 |
| Coronary artery disease  Absent  Present | 2.5 (1.1-8.6)  3.9 (2.4-6.9) | <0.001 |
| Heart failure  Absent  Present | 2.5 (1.1-4.7)  7.6 (5.6-9.1) | <0.001 |
| PVD  Absent  Present | 2.5 (1.1-4.8)  9.6 (3.4-21.4) | 0.01 |
| Macrovascular complications  Absent  Present | 2.5 (1.1-4.7)  3.9 (2.4-6.9) | <0.001 |
| Psychiatric disorders  Absent  Present | 2.5 (1.1-4.8)  3.1 (1.2-6.9) | 0.2 |
| DM triggers  Noncompliance  Infections  New DM diagnosis  Others  Pancreatitis  Medication supply issue  Iatrogenic  Surgery  Medication side-effects  Fasting  GI bleeding | 1.75 (1-3.1)  2.9 (1.1-6.7)  3.3 (1.8-5.1)  2 (1-3.9)  5.7 (3.1-8.5)  2.45 (1.5-3.8)  1.2 (1-2.4)  4.2 (2.9-7)  1.8 (1-3.3)  1 (1-1.6)  5.8 (4-7) | <0.001 |
| DKA severity  Mild  Moderate  Severe | 2.1 (1.1-4.2)  2.2 (1-4.4)  3 (1.4-5.4) | 0.002 |

**Supplementary Table 5.** Clinical Characteristics of all-cause 90-readmission rate in patients with DKA, based on data from the index DKA admission.

| Variable (at index admission) | Readmission, N (%)  (Total 140) | No readmission N (%)  (Total 782) | Significance |
| --- | --- | --- | --- |
| Age in years, Mean +/- SD | 36.02 +/- 16.49 | 35.9 +/- 14.2 | 0.94 |
| Gender  Male (575)  Female (347) | 89 (15.4)  51 (14.7) | 486 (84.52)  296 985.3) | **0.7** |
| Ethnicities  Arabic (502)  Asian (300)  Africans (90)  Others (30) | 86 (17.1)  37 (12.3)  13 (14.4)  4 (13.3) | 416 (82.87)  263 (87.67)  77 (85.56)  26 (86.67) | 0.3 |
| Ethnicity adjusted BMI (Mean ± SD) | 24.5 +/- 6.1 | 24.94 +/- 6.3 | 0.49 |
| Total Existing DM (598)  Total New DM (324) | 92 (15.3)  48 (14.8) | 506 (84.62)  276 (85.19) | 0.8 |
| Existing T1D (332)  New T1D (148)  Existing T2D (266)  New T2D (176) | 57 (17.17)  22 (14.86)  35 (13.16)  26 (14.77) | 275 (82.83)  126 (85.14)  231 (86.84)  150 (85.23) | 0.59 |
| HbA1c admission (Mean +/- SD) | 11.99 +/- 3.42 | 12.06 +/- 2.64 | 0.81 |
| LOS in days admission, Median (IQR) | 2.4 (1-5.6) | 2.6 (1.2-4.7) | 0.63 |
| DKA duration, Median (IQR) | 17 (11-28) | 18 (10.21-29) | 0.61 |
| Hgb at discharge (Mean +/- SD) | 11.81 +/- 2.48 | 12.28 +/- 1.83 | 0.052 |
| RBG at discharge (Mean +/- SD) | 11.54 +/- 4.85 | 11.91 +/- 4.66 | 0.41 |
| Microvascular complications of DM (95)  Macrovascular complications of DM (5) | 20 (21)  15 (26.7) | 75 (79)  41 (73.21) | 0.09  0.01 |
| DKA severity  Mild (202)  Moderate (332)  Severe (388) | 34 (16.8)  48 (14.4)  58 (14.9) | 168 (83.17)  284 (85.54)  330 (85.05) | 0.7 |
| CCI  1 (677)  2 (83)  3 (162) | 89 (13.14)  19 (22.89)  32 (19.75) | 588 (86.85)  64 (77.1)  130 (80.25) | 0.01 |
| Non-compliance to insulin (166) | 20 (12.04) | 146 (87.95) | 0.68 |
| Need for admission to MICU (229) | 27 (11.7) | 202 (88.3) | 0.09 |
| Consult to diabetes educator (348)  Not consulted to diabetes educator (574) | 44 (12.64)  96 (16.72) | 304 (87.35)  478 (83.27) | 0.09 |

(CCI: Charleson comorbidity index, CCI Categorization: CC index 1=1, 2=2, 3-1=3, HbA1c: Glycated haemoglobin, RBG: Random blood glucose, WBC: White blood cell count, Hgb: Haemoglobin, Plt: Platelets, ANC: Absolute neutrophil count, LC: lymphocyte count)

**Supplementary Table 6.** Univariate analysis of factors associated with a 6-month recurrence of DKA following the index DKA admission.

| Characteristics (N) | No recurrence (849) | Recurrence (73) | Significance |
| --- | --- | --- | --- |
| Age (mean +/- SD) | 36.1 +/- 14.6 | 34 +/- 14.4 | 0.1 |
| Male (575)  Female (347) | 534 (92.8)  315 (90.7) | 41 (7.1)  32 (9.2) | 0.2 |
| Ethnicity  Arabic (502)  Asian (300)  African (90)  Others (30) | 457 (91)  281 (93.6)  83 (92.2)  28 (93.3) | 45 (8.9)  19 (6.3)  7 (7.7)  2 (6.6) | 0.6 |
| DM diagnosis  Pre-existing DM (598)  New DM (324)  Pre-existing T1D(332)  Pre-existing T2D (266)  New T1D (148)  New T2D (176) | 536 (89.6)  313 (96.6)  289 (87)  247 (92.8)  139 (93.9)  174 (98.8) | 62 (10.3)  11 (3.4)  43 (12.9)  19 (7.1)  9 (6)  2 (1.1) | <0.001  <0.001 |
| Weight (mean +/- SD) | 67.1 +/- 20.1 | 63.1 +/- 18.3 | 0.051 |
| BMI (mean +/- SD) | 24.9 +/- 6.3 | 23.8 +/- 5.7 | 0.08 |
| Microvascular complications  Absent (827)  Present (95) | 759 (91.7)  90 (94.7) | 68 (8.2)  5 (5.2) | 0.3 |
| CC index  1 (677)  2 (83)  3 (80)  4 (28)  5 (22)  6 (16)  7 (7)  8 (4)  9 (2)  10 (2)  11 (1) | 623 (92)  73 (87.9)  75 (93.7)  26 (92.8)  22 (100)  16 (100)  6 (85.7)  3 (75)  2 (100)  2 (100)  1 (100) | 54 (7.9)  10 (12)  5 (6.2)  2 (7.1)  0  0  1 (14.2)  1 (25)  0  0  0 | 0.6 |
| Macrovascular complications  Absent (866)  Present (56) | 795 (91.8)  54 996.4) | 71 (8.2)  2 93.5) | 0.2 |
| COVID-19 infection  Absent (911)  Present (11) | 839 (91.9)  11 (100) | 73 (8)  0 | 0.3 |
| DM triggers  Non-compliance (262)  Infections (241)  New DM diagnosis (223)  Others (86)  Pancreatitis (25)  Medication supply issue (24)  Iatrogenic (24)  Surgery (13)  Medication side-effects (8)  Fasting (9)  GI bleeding (6) | 232 (27.3)  224 (26.4)  215 (25.3)  76 (8.9)  22 (2.5)  24 (2.8)  22 (2.5)  12 (1.4)  7 (0.8)  8 (0.9)  6 (0.7) | 30 (41.1)  17 (23.2)  8 (10.9)  10 (13.7)  3 (4.1)  0  2 (2.7)  1 (1.3)  1 (1.3)  1 (1.3)  0 | 0.1 |
| DKA severity  Mild (202)  Moderate (332)  Severe (388) | 189 (93.5)  306 (92.1)  354 (91.2) | 13 (6.4)  26 (7.8)  34 (8.7) | 0.6 |
| HbA1c (%), Mean +/- SD | 12.08 +/- 2.7 | 11.6 +/- 3 | 0.18 |
| LOS (days)  Mean +/- SD | 4.5 +/- 7.9 | 3.7+/-5.6 | 0.2 |
| MICU admission (229) | 218 (25.6) | 11 (15) | 0.04 |

**Supplementary Table 7.** Univariate analysis of factors predicting 12-month recurrence of DKA from the index admission.

| Characteristics (N) | No recurrence (861) | Recurrence (61) | Significance |
| --- | --- | --- | --- |
| Age (mean +/- SD) | 36.3 +/- 14.6 | 30 +/- 12.8 | 0.001 |
| Male (575)  Female (347) | 552 (96)  309 (89) | 23 (4)  38 (10.9) | <0.001 |
| Ethnicity  Arabic (502)  Asian (300)  African (90)  Others (30) | 454 (90.4)  291 (97)  87 (96.6)  29 (96.6) | 48 (9.5)  9 (3)  3 (3.3)  1 (3.3) | 0.001 |
| DM diagnosis  Total T1D (480)  Total T2D (442)  Pre-existing DM (598)  New DM (324)  Pre-existing T1D(332)  Pre-existing T2D (266)  New T1D (148)  New T2D (176) | 429 (89.3)  432 (97.7)  541 (90.4)  320 (98.7)  285 (85.8)  256 (96.2)  144 (97.3)  176 (100) | 51 (10.6)  10 (2.2)  57 (9.5)  4 (1.2)  47 (14.1)  10 (3.7)  4 (2.7)  0 | <0.001  <0.001  <0.001 |
| Weight (mean +/- SD) | 67.2 +/- 20.07 | 61.8 +/- 18.6 | 0.04 |
| BMI (mean +/- SD) | 24.9 +/- 6.3 | 24.4 +/- 6.1 | 0.5 |
| Microvascular complications  Absent (827)  Present (95) | 770 (93.1)  91 (95.7) | 57 (6.8)  4 (4.2) | 0.3 |
| CC index  1 (677)  2 (83)  3 (80)  4 (28)  5 (22)  6 (16)  7 (7)  8 (4)  9 (2)  10 (2)  11 (1) | 630 (73.1)  76 (8.8)  74 (8.5)  28 (3.2)  22 (2.5) 16 (1.8)  7 (0.8)  3 (0.3)  2 (0.2)  2 (0.2)  1 (0.1) | 47 (77)  7 (11.4)  6 (9.8)  0  0  0  0  8 (1.6)  0  0  0 | 0.5 |
| Macrovascular complications  Absent (866)  Present (56) | 808 (93.3)  53 (94.6) | 53 (6.7)  3 (5.3) | 0.6 |
| COVID-19 infection  Absent (911)  Present (11) | 850 (93.3)  11 (100) | 61 (6.7)  0 | 0.3 |
| DM triggers  Non-compliance (262)  Infections (241)  New DM diagnosis (223)  Others (86)  Pancreatitis (25)  Medication supply issue (24)  Iatrogenic (24)  Surgery (13)  Medication side-effects (8)  Fasting (9)  GI bleeding (6) | 237 (27.5)  225 (26.1)  222 (25.8)  74 (8.6)  25 (2.9)  23 (2.6)  21 (2.5)  12 (1.4)  7 (0.8)  8 (0.9)  6 (0.7) | 25 (40.9)  16 (26.2)  1 (1.6)  12 (19.6)  0  1 (1.6)  3 (4.9)  1 (1.6)  1 (1.6)  1 (1.6)  0 | 0.001 |
| DKA severity  Mild (202)  Moderate (332)  Severe (388) | 192 (95)  307 (92.4)  362 (93.3) | 10 (4.9)  25 (7.5)  26 (6.7) | 0.5 |
| HbA1c (%), Mean +/- SD | 12.06+/-2.7 | 11.8 +/- 2.7 | 0.6 |
| LOS (days)  Mean +/- SD | 4.6 +/- 7.9 | 2.3 +/- 1.8 | 0.02 |
| MICU admission  Absent (693)  Present (229) | 645 (93)  216 (94.3) | 48 (6.9)  13 (5.6) | 0.5 |
| 6-month recurrence  Absent (849)  Present (73) | 827 (97.4)  34 (46.5) | 22 (2.5)  39 (53.4) | <0.001 |
| Consult to DE (348)  Present (348)  Absent (574) | 341 (98)  520 (90.6) | 7 (2)  54 (9.4) | <0.001 |
